# Commentary: Caring for learners – humanising clinical education to build compassionate and competent nurses

**DOI:** 10.1177/17449871261465551

**Published:** 2026-08-29

**Authors:** Paulo Seabra

**Affiliations:** University of Lisbon, School of Nursing, Nursing Research Innovation and Development Centre of Lisbon (CIDNUR), Lisboa, Portugal

**Commentary to:** Empowering future nurses – exploring the relationship between higher education-related stress, academic self-efficacy and clinical education experience satisfaction among nursing students in Saudi Arabia: a cross-sectional study (Mohammed Hussien, 2026).

This study explores a pivotal milestone in nursing education: the complex, high-stakes transition where students leave the controlled safety of theoretical classrooms to face the unpredictable realities of real-world patient care. Utilising a robust analytical framework, the research examines how the unique stresses of higher education and a student’s academic self-efficacy – their core belief in their own professional capabilities – interlock to dictate overall satisfaction with clinical training. Grounded in the urgent workforce development and localisation goals of Saudi Vision 2030 (culturally sensitive), this study serves as an essential empirical starting point. Its primary mission helps nursing faculties design institutional policies that actively protect the psychological well-being of future healthcare professionals while maximising the pedagogical value of their practical training across hospital placements.

## Key findings

The weight of the environment and human relations (stress): Clinical training takes place against a heavy, constant backdrop of environmental stress. However, the study’s most profound revelation challenges a traditional misconception: academic burnout among student nurses is not primarily driven by the volume of technical procedures or the medical complexity of patients. Instead, the real vulnerability stems from ‘interface stress’. This concept captures the severe anxiety triggered by interpersonal friction within the clinical and academic ecosystem. Students feel profoundly burdened by horizontal communication barriers with hospital staff, the intimidation of constant observation and the paralysing fear of making mistakes in front of evaluators. When personal life hardships – such as financial anxiety, poor sleep or a lack of time for self-care – merge with this interpersonal pressure, a cumulative domino effect severely damages the student’s psychological stability and clinical focus. Ultimately, these dynamics act as direct catalysts for diminished mental health, closely mirroring the systemic stressors and psychological distress consistently documented in occupational studies of professional nurses (Seabra et al., 2026).

Self-efficacy as a psychological buffer: Academic self-efficacy – the internal confidence to master complex skills, manage emotions and navigate social dynamics – emerges as the student’s ultimate shield. The research highlights a clear behavioural pattern: when students possess high self-efficacy, they develop stronger self-regulation and emotional control. This cognitive strength allows them to reframe heavy clinical demands as exciting professional challenges rather than terrifying threats to their identity. However, this shield is not bulletproof. When interface stress and personal hardships exceed a student’s resilience threshold, the cognitive buffer shatters, leaving them fully exposed to severe learning fatigue, acute burnout and a loss of professional motivation.

The multifactorial dynamics of satisfaction: What truly makes a student look back on their clinical experience with satisfaction? The data prove that true satisfaction transcends modern hospital infrastructure or perfect curriculum alignment. At the very heart of a successful clinical rotation lie empathy and psychological safety. Students report the highest levels of satisfaction when they feel that their clinical instructors and preceptors validate their emotions, remain approachable for constructive feedback and offer a supportive mentoring stance. A positive, symbiotic loop is revealed: high self-confidence fuels clinical satisfaction, and a satisfying, humanised environment, in turn, mitigates stress. Conversely, a hostile or indifferent ward atmosphere causes satisfaction to plummet, directly threatening long-term student retention (Bakker et al., 2020).

## Critical insights for education

These findings demand an intentional, compassionate shift in how nursing faculties and hospital networks co-design practical training:

Humanising clinical preceptorship: Clinical instructors must actively move away from rigid, fear-based or purely coercive evaluation models. As preceptors hold the keys to a student’s psychological safety, they must foster a learning culture where clinical errors are treated as valuable opportunities for reflective growth and clinical reasoning, rather than grounds for emotional punishment (Selim et al., 2024).
Integrating emotional competencies and re-engineering simulation: Academic curricula cannot afford to focus solely on technical skill acquisition. Training in emotional intelligence, conflict resolution, self-care and stress-coping mechanisms must be formally woven into the coursework. Furthermore, high-fidelity simulation must expand beyond medical emergencies to incorporate ‘interpersonal stress tests’ – such as navigating difficult communication barriers and handling critical feedback under pressure – to prepare students emotionally before they enter a hospital ward. In addition, low-fidelity simulation, but with a high degree of personal attention to detail, will always be a good feature.

## Critical insights for future research

Methodologically, the study’s cross-sectional nature captures a single, static snapshot of a highly fluid human experience, preventing direct causal proofs. Future research should adopt longitudinal models to track how stress and confidence fluctuate as a student progresses across different academic years and into their final residency (Bakker et al., 2020). Additionally, given Saudi Arabia’s unique socio-educational landscape – characterised by single-gender learning environments and distinct cultural norms guiding patient–student interactions – future studies must explore how local dynamics influence a student’s willingness to seek out institutional mental health support.

## Theoretical innovation and future contribution

The true innovation of this research lies in its holistic approach. Rather than analysing stress, confidence and satisfaction as isolated pieces, the study maps a powerful psychological chain reaction: interpersonal and personal stressors directly erode a student’s self-confidence, tripping a wire that floods their clinical experience with dissatisfaction. This vital insight shifts the burden of blame away from individual student flaws. It demonstrates that student burnout is not a character defect or a lack of personal grit; it is a structural vulnerability built into the very design of healthcare education environments. Faced with a critical global nursing shortage, the battle to retain talent must be fought and won within academia. Building compassionate, communicative and structured mentorship bridges between universities and healthcare institutions is the only viable pathway to cultivating professionally secure, emotionally resilient nurses who possess the strength to deliver high-quality, compassionate patient care.
